# Mucopolysaccharidosis Type VI with Recurrent Chest Infection

**DOI:** 10.7759/cureus.35229

**Published:** 2023-02-20

**Authors:** Ashraf Numan, Anoud N Alruwaili, Rehab Ali, Hamasat Alsharari, Mishal Alanazi, Nouf N Alazmi, Ahmed A Alsaati

**Affiliations:** 1 Paediatrics, Alqurayyat General Hospital, Alqurayyat, SAU; 2 Medicine and Surgery, Al Jouf University, Sakaka, SAU; 3 General Practice, Alqurayyat General Hospital, Alqurayyat, SAU; 4 Medicine and Surgery, King Faisal University, Al-Hofuf, SAU

**Keywords:** pediatrics, chest infection, deformities, genetics, syndromic diseases, autosomal recessive disease, mucopolysaccharidosis

## Abstract

Mucopolysaccharidosis type VI (Maroteaux-Lamy syndrome) is a progressive multi-systemic autosomal recessive disease resulting from a deficiency of arylsulfatase B (N-acetylgalactosamine-4-sulfatase). Here we report the case of a three-year-old male child born full-term via normal vaginal delivery. He had frequent admissions due to a chest infection that started at two months of age. At the age of 23 months, he was admitted after complaining of shortness of breath (SOB) due to asthma and aspiration pneumonia; additionally, dysmorphic features were noticed (single palmar crease, short round toes, coarse facial features such as a flat nose, big lips).

A genetic study showed mucopolysaccharidosis VI (MPS VI). At three years of age, he was complaining of cough and SOB. Examination showed wheezing all over the chest, normal first and second heart sounds (S1 and S2), a murmur with no clicks, hepatosplenomegaly, and a palpable left kidney. However, the central nervous system (CNS) and eye examinations were normal. Echocardiography revealed a thickened bicuspid aortic valve, mild aortic regurgitation, and mitral regurgitation. Therefore, the patient presented with different clinical symptoms of MPS VI.

It is important to increase the physicians' awareness about MPS by focusing on increasing the probability of MPS as a differential diagnosis whenever patients present with abnormal appearance, limb deformities, and recurrent unexplained infections; hence, making early diagnosis and treatment decisions, leading to a slowing down of the progression of the disease and enhancing the patient's quality of life.

## Introduction

Mucopolysaccharidosis (MPS) is a collection of genetic mistakes in complex chemical catabolism, mostly affecting patients in their childhood, that comes in different forms that are caused by enzyme deficits and result in different clinical symptoms for each form [1-4]. Mucopolysaccharidosis VI (MPS VI), also known as Maroteaux-Lamy syndrome, is a progressive multi-systemic lysosomal storage disease that is autosomal recessive and caused by a lack of arylsulfatase B (ARSB), which causes dermatan sulfate to build up in the body, leading to the degradation of glycosaminoglycans (GAG), which is caused by mutations in the ARSB gene. It was first described by French doctors Pierre Maroteaux and Maurice Lamy in 1963 [5-11].

Initially, children with MPS VI typically present in a healthy state at birth, with symptoms typically emerging within the first few months of life as a result of elevated GAG concentrations in cells. Although MPS VI is normally categorized as either slowly advancing or rapidly moving depending on the severity of the symptoms, it is now known to have an intermediate form of MPS between the two [12]. Patients with MPS VI experience a variety of multi-systemic symptoms over a typically progressive and chronic course that primarily affect their cardiovascular, respiratory, and skeletal systems, as well as their skin, cornea, liver, spleen, meninges, and brain. In addition to these diseases, they can also affect health-related quality of life and activities of daily living [1,5,6,13]. In general, examining the excretion of GAGs in the urine is typically the first step in the diagnosis of MPS VI. X-ray, ultrasound, computed tomography (CT), and magnetic resonance imaging (MRI) are routinely utilized diagnostic techniques for skeletal, cardiac, and cerebral involvements, as well as genetic studies [2,14].

Up until recently, the only treatment options for MPS VI were supportive therapy, bone marrow transplantation therapies, and enzyme replacement therapy (ERT). In addition to symptomatic treatment of complications like the use of oxygen with positive pressure while sleeping, tonsillectomy, adenoidectomy, tracheostomy, medical or surgical treatment for heart failure, cervical decompression, corneal transplantation, and ventricular shunt, among others, supportive therapy focuses on nutrition, occupational therapy, and physical therapy [14]. For quick, targeted treatment with enzyme replacement therapy (ERT), which is now offered for MPS types I, II, IV, and VI, early identification is crucial. An early therapy subsequently reduces the building up of intracellular GAGs and slows down the progression of multi-organ dysfunction, hence improving lung and heart function and resulting in an accelerated growth rate [3].

The patient's prognosis is likely influenced by the patient's age at the onset of the initial symptoms, the rate of the disease's advancement, the age at which enzyme replacement therapy or hematopoietic stem cell transplantation treatment was initiated, and the medical and surgical team's experience. Patients with both fast and slowly advancing forms of the disease could sustain permanent harm if the condition continued unrecognized and untreated and enzyme replacement therapy was subsequently postponed [7]. Worldwide improvements have been made in MPS treatment; however, due to a paucity of enzymes and molecular tests in low- and middle-income countries, it is challenging to confirm a diagnosis of one of the MPS [15].

## Case presentation

The patient was a three-year-old Saudi male child born after a normal vaginal delivery and multiple admissions due to recurrent complaints. When he was two months old, he was admitted for the first time due to nasal obstruction, cough, and shortness of breath, and was later diagnosed with bronchiolitis and pneumonia. On his second admission, at seven months, he returned with similar complaints but a developmental delay as his neck was lax with a wide open anterior fontanel. When he was a year old, he came back due to more complaints of nasal obstruction, snoring, nasal and postnasal discharges, and enlarged tonsils. A CT scan done during the visit showed adenoid hypertrophy. In addition, an X-ray confirmed the existence of a thick wrist. At 15 months, he was admitted once again with complaints of respiratory distress, and his examination showed a pigeon chest with crowded ribs, hepatosplenomegaly of 4 centimeters (cm) under the costal margin, finger clubbing, abdominal laxity, and an umbilical hernia. At 23 months, he was admitted with shortness of breath and was found to be asthmatic, with aspiration pneumonia in the right lung and a mass in the subcostal region, hepatosplenomegaly, and dimorphic features in the form of coarse facial features, a flat nose, big lips, a single palmar crease, and short toes. Therefore, a genetic study was done, showing mucopolysaccharidosis type VI. However, his echocardiography was normal. In later admissions, he came for multiple complaints of asthma attacks with right upper lobe collapse in the chest x-ray. Ultimately, when he was three years old, he still had complaints of cough and shortness of breath; only this time, however, he developed a new sign of murmur during auscultation. Upon examination, the patient was conscious, febrile, ill-looking, hypoactive, dimorphic, and distressed with noisy breathing. The patient was not jaundiced or cyanosed and wasn’t pale either. His weight was 14 kilograms (kg), and he was 90 cm tall. Upon facial examination, the patient had multiple dysmorphic features, such as a coarse face, a flat nose, and big lips. Additionally, he had noticeable lymph node enlargements on the neck. On chest examination, there were equal bilateral air entries, but wheezes were heard all over the chest. Upon cardiac examination, both S1 and S2 heart sounds were normal, and there was a murmur without any clicks. Lastly, an abdominal examination showed hepatosplenomegaly and a palpable left kidney, but the CNS and eye examinations were normal.

Laboratory investigations were done during different admissions. At the time of diagnosis, a complete blood count (CBC) showed low hemoglobin of 10 grams per deciliter (g/dl), low eosinophils of 0,19, and low hematocrit of 32.5%, while mean platelet volume was high at a level of 9.1 femtoliters (FL). However, other laboratory components were within normal ranges. Furthermore, the fasting glucose level was too high, reaching 6.7 millimoles per liter (mmol/L), serum electrolytes were within normal limits, and liver and renal function tests were normal. but the alkaline phosphatase enzyme was too high at 298 international units per liter (IU/L), and the thyroid function test (TFT) was normal [Table 1].

**Table 1:** Laboratory results of the patient Gram per deciliter (g/dl), cells per microliter (cells/μl), percentage (%), femtoliter (FL), millimoles per liter (mmol/L), and international units per liter (IU/L)

Investigations	Results	Units	Reference range
Hemoglobin	10	g/dl	11.2 to 14.5
Eosinophils	0,19	Cells\mcL	More then 700
Hematocrit	32.5	%	35 to 44 %
Mean platelets volume	9.1	FL	
Fasting glucose level	6.7	mmol/L	5.0 to 10.0
Electrolytes	Normal		
Alkaline phosphatase	298	IU/L	44 to 147

Blood culture and methicillin-resistant Staphylococcus aureus (MRSA) screenings were negative. Moreover, the genetic study confirmed the diagnosis. His abdominal ultrasound showed hepatosplenomegaly, while his echocardiography showed situs solitus, concordant connections, an intact interatrial septum, a thickened bicuspid aortic valve, mild aortic regurgitation, and mitral regurgitation; however, there were no ventricular septal defects (VSD), patent ductus arteriosus (PDA), aortic stenosis, ventricular hypertrophy, left ventricular outflow tract obstruction (LVOT), nor right ventricular outflow tract obstruction (RVOT).

**Figure 1:**
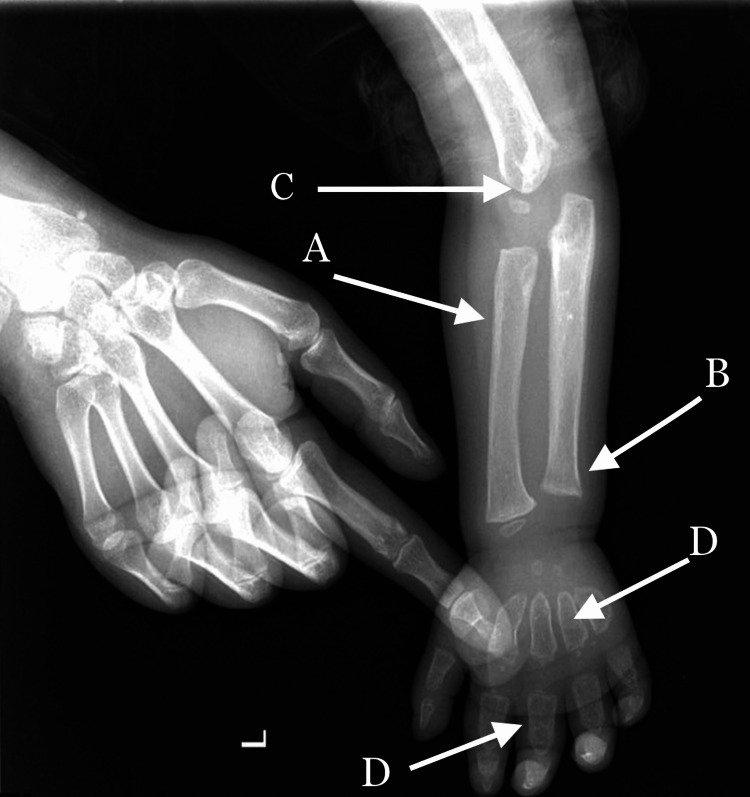
X-ray of the forearm Short diaphysis [A], flattened epiphysis [B], flamed upper limb bones [C], and bullet-shaped metacarpal bones [D]

A forearm x-ray showed a wide, short diaphysis, flattened epiphysis flares of upper limb bones, and bullet-shaped metacarpal bones [Figure 1].

**Figure 2:**
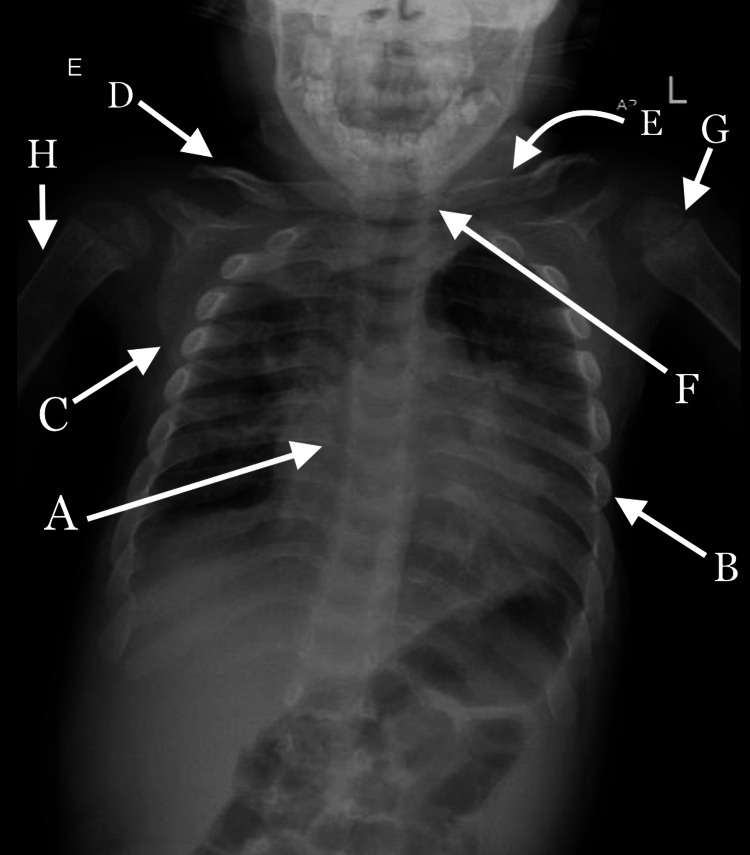
Chest X-ray Ribs narrowing on the sternum end [A], ribs widening on the vertebral end [B], dysplastic scapula [C], hypoplastic on the lateral end of the clavicle [D], broadening the clavicle [E], short neck [F], flattened epiphysis [G], wide short diaphysis [H]

The musculoskeletal system’s X-ray showed narrowing of the ribs on the sternum’s end and widening on the vertebral end, scapular dyskinesis, hypoplasia of the lateral end of the clavicle, broadening clavicles, and a short neck [Figure 2].

The patient received enzyme replacement therapy (ERT), as well as conservative treatment by giving the patient antibiotics, oxygen inhalation with nebulizers, IV fluids, and treatment of the underlying conditions.

## Discussion

In this report, we present a case of MPS VI in which the patient was suspected to be suffering from mucopolysaccharidosis disorder based on the clinical pictures. The patient had multiple recurrent admissions to the hospital due to respiratory problems, which was the key point in forming the definitive diagnosis. Furthermore, the boy had suffered from multiple attacks of asthma and chest infections, his tonsils were enlarged, and his CT showed adenoid hypertrophy as well as right upper lobe collapse in both the chest X-ray and the CT scan. Previous studies revealed that both upper and lower airway obstructions can lead to recurrent upper and lower respiratory tract infections, which are typical features of MPS [16,17].

In this current study, the patient was normal at birth; however, his symptoms started in his first year of life. Most previous studies reported that the onset age was commonly around the first or second year of age, except for MPS VII [15]. Our patient had a thickened bicuspid aortic valve, mild aortic regurgitation, and mitral regurgitation; these abnormalities within the cells of the heart and its functions may be explained by GAG accumulation. Previous studies showed that echocardiography can detect valvular heart diseases, with the mitral valve accounting for 96%, the tricuspid valve for 71%, and the aortic valve for 43%. Therefore, cardiac evaluations by electrocardiography, echocardiography, and blood pressure readings were recommended every one to two years to assess changes in the heart’s structure, function, rhythm, and conduction [15,18].

The patient presented with different clinical symptoms of MPS VI, which were mostly related to skeletal deformities such as short stature, pigeon chest with crowded ribs, mobile joints, scapular dyskinesis, hypoplasia of the lateral end of the clavicle, broadening of the clavicle, short neck, as well as wide and short diaphyses, flattened epiphyses flared on upper limb bones, and bullet-shaped metacarpal bones. The previous literature suggested that GAG accumulation in articular cartilage promotes inflammation and chondrocyte apoptosis, which leads to the release of matrix metalloproteinases (MMPs), which subsequently leads to progressive degenerative joint disease [19,20].

## Conclusions

Although there is a high prevalence of MPS in Saudi Arabia compared to other countries, it remains an underestimated medical challenge that needs an earlier diagnosis as well as prompt intervention; therefore, it is important to increase doctors' awareness about MPS, particularly the attenuated form, and increase suspicion of MPS when a patient is presented with an abnormal appearance (short stature, a coarse face, a wide nose, a short neck, a short nasal bridge, and limb deformities) and with significant clinical signs and symptoms, such as recurrent respiratory and ear symptoms as well as mental retardation. Therefore, early diagnosis and treatment lead to slowing down the disease’s progression, eventually enhancing the patient's quality of life.
